# Combined repetitive transcranial magnetic stimulation and transcutaneous spinal cord stimulation for upper limb recovery in chronic incomplete cervical spinal cord injury: protocol for a pilot randomized controlled trial

**DOI:** 10.3389/fnins.2026.1833493

**Published:** 2026-05-28

**Authors:** Ravi Shankar, Ning Tang, Gobinathan Chandran

**Affiliations:** 1 Clinical Research & Innovation Office, Tan Tock Seng Hospital, National Healthcare Group, Singapore, Singapore; 2 Department of Medicine, Division of Rehabilitation Medicine, National University Hospital, National University Health System, Singapore, Singapore; 3 Department of Rehabilitation, Alexandra Hospital, National University Health System, Singapore, Singapore; 4 Yong Loo Lin School of Medicine, National University of Singapore, Singapore, Singapore

**Keywords:** cervical spinal cord injury, convergent neuromodulation, intermittent theta burst stimulation, neuromodulation, neuroplasticity, pilot randomized controlled trial, repetitive transcranial magnetic stimulation, tetraplegia

## Abstract

**Background:**

Cervical spinal cord injury (SCI) results in severe upper limb impairment, with restoration of hand and arm function ranked as the highest rehabilitation priority by individuals with tetraplegia. Transcutaneous spinal cord stimulation (tSCS) has emerged as a promising approach for enhancing upper limb recovery. Intermittent theta burst stimulation (iTBS), an efficient form of repetitive transcranial magnetic stimulation, can enhance cortical excitability and descending motor drive. However, the benefit of combining these complementary neuromodulation modalities to simultaneously target supraspinal and spinal circuits has not been evaluated in a controlled trial.

**Objective:**

This study aims to evaluate the feasibility, safety, and preliminary efficacy of combined cortical neuromodulation (iTBS) and spinal neuromodulation (tSCS) versus tSCS alone, each paired with standardized upper limb rehabilitation, for improving upper limb motor function in chronic incomplete cervical SCI.

**Methods:**

This single-center, two-arm, assessor-blinded, pilot randomized controlled trial will enroll 24 adults aged 21–65 years with chronic (more than 12 months post-injury) incomplete cervical SCI (American Spinal Injury Association Impairment Scale grade C or D, neurological level C2 to C8) at Alexandra Hospital, Singapore. Participants will be randomized 1:1 to receive either iTBS combined with tSCS plus standardized upper limb rehabilitation or tSCS plus upper limb rehabilitation alone. Interventions will be delivered twice weekly for 12 weeks (24 sessions). The primary outcome is the change in Upper Extremity Motor Score from baseline to 12 weeks. Secondary outcomes include measures of upper limb function, independence, spasticity, corticospinal excitability, quality of life, and goal attainment. Assessments will be conducted at baseline, post-intervention, and at 4- and 12-week follow-up.

**Results:**

This protocol was approved by the National Healthcare Group Domain Specific Review Board. Recruitment is expected to begin in the third quarter of 2026, with data collection anticipated to be completed by the fourth quarter of 2027.

**Conclusion:**

This pilot trial will provide the first controlled evidence on whether adjunctive cortical neuromodulation via iTBS produces additional upper limb motor recovery beyond tSCS-based rehabilitation in chronic incomplete cervical SCI. Feasibility data and effect size estimates will inform the design of a subsequent multicenter confirmatory trial.

**Clinical trial registration:**

ClinicalTrials.gov, identifier NCT07586644.

## Introduction

### Background

Spinal cord injury (SCI) is a devastating neurological condition that disrupts motor, sensory, and autonomic function, leaving millions worldwide with chronic disability. The World Health Organization estimates that SCI affects approximately 15.4 million people globally (Lu et al., 2025). Among those with cervical-level injuries, which account for roughly half of all traumatic SCI cases globally (Safdarian et al., 2023), impairment of upper extremity (UE) function profoundly limits independence in activities of daily living and quality of life (Ahuja et al., 2017; van Silfhout et al., 2017). Restoration of hand and arm function is consistently identified as the highest rehabilitation priority by individuals with tetraplegia, surpassing even recovery of locomotion and bladder control (Anderson, 2004). Despite advances in conventional rehabilitation approaches, including physical therapy, occupational therapy, and activity-based training, functional recovery of the upper limb in the chronic phase of cervical SCI remains limited, highlighting a critical unmet clinical need (James et al., 2018; Sayenko et al., 2015).

The pathophysiological basis of motor impairment in incomplete cervical SCI involves disruption of descending corticospinal and other supraspinal motor pathways, alongside reorganization of spinal interneuronal circuits below the level of injury (Anderson et al., 2022; Zholudeva et al., 2018). Critically, incomplete SCI is characterized by the preservation of residual neural pathways bridging the lesion site, representing a biological substrate for experience-dependent and stimulation-driven neuroplasticity (Hutson and Di Giovanni, 2019). It is increasingly recognized that neuroplasticity is activity-dependent, suggesting that external neuromodulatory stimuli may enhance recovery after SCI when paired with structured rehabilitation (Barss et al., 2022; Behrman et al., 2006; Dietz and Fouad, 2014; Raineteau and Schwab, 2001; Zheng et al., 2020). This understanding has catalyzed the development of novel non-invasive neurostimulation strategies targeting both cortical and spinal levels of the motor neuraxis.

### Transcutaneous spinal cord stimulation

Transcutaneous spinal cord stimulation (tSCS) is a non-invasive neuromodulation technique that delivers electrical stimulation through surface electrodes positioned over the posterior aspect of the spine. Typically employing biphasic rectangular waveforms with carrier frequencies of 5–10 kHz delivered at 30–70 Hz, tSCS activates large-to-medium diameter sensory afferent fibers in the dorsal roots, which supply excitatory synaptic connections to motoneurons and spinal interneuronal circuits (Hofstoetter et al., 2020; Sayenko et al., 2015). Seminal work by Gerasimenko et al. (2015) demonstrated that tSCS can enable voluntary movements in individuals with motor-complete SCI, providing foundational evidence for spinal circuit reactivation. Subsequent investigations have confirmed the feasibility and preliminary efficacy of tSCS for improving motor function, standing ability, and walking parameters in individuals with incomplete injuries (Hofstoetter et al., 2021; Taccola et al., 2018).

A systematic review by Tajali et al. (2024), published in Frontiers in Neuroscience in 2024, demonstrated that tSCS modulates both spinal and corticospinal excitability, with studies showing increased motor evoked potentials and decreased H-reflex amplitudes following alternating current tSCS, indicating enhanced neuroplasticity at multiple levels of the motor system.

The strongest clinical evidence for cervical tSCS comes from the Up-LIFT trial, a prospective, single-arm, multicenter study published in Nature Medicine in 2024 by Moritz et al. (2024). This trial enrolled 60 participants with chronic cervical SCI at 14 international centers and demonstrated that non-invasive spinal cord electrical stimulation (ARCEX Therapy) combined with structured rehabilitation significantly improved upper limb strength, function, and sensation compared to rehabilitation alone. The study reported that 72% of participants met the primary effectiveness endpoint, with significant improvements in fingertip pinch force, hand prehension, UE motor and sensory scores, and self-reported quality of life. No serious adverse events related to stimulation were observed. In December 2024, the ARC-EX System received FDA *de novo* authorization, marking the first FDA-cleared non-invasive spinal cord stimulation system for SCI (Mertz, 2025).

Subsequent studies have extended these findings. A home-based extension of the Up-LIFT trial, published in Neurology Clinical Practice in 2025, demonstrated that tSCS can be safely and effectively delivered in the home environment (Tefertiller et al., 2025). Programming recommendations based on analysis of stimulation parameters from both the Up-LIFT and LIFT Home trials were published in Neuromodulation in 2025, providing a clinical framework for parameter selection (Gelenitis et al., 2025). A real-world pilot study by Suggitt et al. (2025), published in Neuromodulation in 2025, confirmed the safety and effectiveness of multisite tSCS combined with activity-based therapy in a community rehabilitation setting. A comprehensive review by Singh et al. (2024) in the Journal of Neurotrauma in 2024 synthesized the evidence for cervical tSCS in UE recovery and concluded that, although the evidence base is limited in sample size, findings consistently suggest that tSCS combined with task-specific training improves voluntary control of arm and hand function. A systematic review by Wankner et al. (2026), published in Life in 2026, further synthesized preclinical and clinical evidence across epidural, intraspinal, and transcutaneous modalities and confirmed the therapeutic promise of cervical spinal cord stimulation while emphasizing the need for standardized protocols and larger controlled trials.

### Repetitive transcranial magnetic stimulation

Repetitive transcranial magnetic stimulation (rTMS) is a non-invasive brain stimulation technique that modulates cortical excitability through pulsed magnetic fields generated by a coil positioned over the scalp (Rossini et al., 2015). When applied at high frequencies or in patterned protocols over the primary motor cortex (M1), rTMS increases corticospinal excitability and has been shown to promote motor recovery in various neurological conditions. The therapeutic mechanisms are believed to involve enhancement of descending corticospinal drive, promotion of cortical reorganization, and induction of long-term potentiation (LTP)-like plasticity at cortical synapses (Fan et al., 2025).

Intermittent theta burst stimulation (iTBS) is a patterned rTMS protocol that delivers bursts of 3 magnetic pulses at 50 Hz, repeated at a theta frequency of 5 Hz, producing facilitatory after-effects on cortical excitability that last well beyond the stimulation period (Huang et al., 2005). A key practical advantage of iTBS is its brevity: a standard 600-pulse session is completed in approximately 3–4 min, compared with 10–20 min for conventional high-frequency rTMS protocols. We recognize, however, that current iTBS evidence derives predominantly from research settings rather than routine clinic use. Its short duration and portable equipment footprint make clinical translation plausible, and this pilot will prospectively capture setup time, therapist burden, and equipment turnover data to directly assess scalability in routine rehabilitation workflows, which has not yet been established.

A comprehensive review of rTMS for SCI, published in the International Journal of Molecular Sciences in January 2025, summarized the current evidence and concluded that rTMS has demonstrated therapeutic effectiveness in increasing corticospinal transmission, enhancing voluntary motor output, improving motor function, alleviating spasticity, and reducing neuropathic pain in individuals with SCI (Fan et al., 2025). The review highlighted iTBS and combined approaches as promising future directions. A feasibility study by Gharooni et al. (2018) examined iTBS for upper limb dysfunction and spasticity in SCI and demonstrated that it was safe and well tolerated, supporting its use in larger clinical trials. More recently, Feng et al. (2023) conducted a pilot randomized controlled trial of cerebral theta-burst stimulation combined with physiotherapy in patients with incomplete SCI and demonstrated significant improvements in lower limb motor scores, walking speed, stride length, and functional independence measures. Shi et al. (2026) further confirmed the efficacy of excitatory iTBS on lower limb recovery in chronic incomplete SCI. A recently published protocol in JMIR Research Protocols by Patel et al. (2025) described a five-arm randomized controlled trial of iTBS for sensorimotor recovery in complete SCI, underscoring the growing research interest in this technique for the SCI population.

### Rationale for combined stimulation: convergent neuromodulation

While both tSCS and rTMS have independently shown promise, neither modality alone addresses the full complexity of the disrupted corticospinal neuraxis in cervical SCI. rTMS enhances descending cortical drive from above the lesion, whereas tSCS increases spinal circuit excitability below the lesion. The theoretical rationale for combining these modalities is that simultaneous top-down cortical facilitation and bottom-up spinal priming may produce synergistic or supra-additive effects on motor recovery, a concept termed “convergent neuromodulation” (Benavides et al., 2020; Wang et al., 2023). Simultaneous delivery is required (not sequential) because spike-timing-dependent plasticity depends on iTBS-primed descending volleys arriving at sublesional motoneurons within ∼10 to 20 ms of tSCS-evoked afferent volleys, enabling Hebbian coincidence detection. Sequential delivery dissipates the brief iTBS after-effect window before afferent priming is engaged; standalone modalities leave either descending drive or sublesional excitability sub-threshold.

This concept is supported by several converging lines of evidence. First, paired associative stimulation (PAS) paradigms, which combine TMS with peripheral or spinal stimulation, have been shown to strengthen corticospinal connections through spike-timing-dependent plasticity (STDP) mechanisms. A systematic review of cortico-spinal PAS in SCI published in Expert Review of Medical Devices in 2024 evaluated 10 studies involving 84 patients and found that PAS improved motor outcomes (Martino Cinnera et al., 2024). Second, Pulverenti et al. (2021, 2022) conducted randomized clinical trials pairing TMS with transcutaneous transspinal stimulation during locomotor training in individuals with chronic SCI, demonstrating neurophysiological changes indicative of spinal synaptic strengthening across multiple segments. Third, the systematic review by Tajali et al. (2024) explicitly called for more rigorous studies comparing the effects of combined neuromodulation versus single techniques on functional and neural recovery after SCI. Fourth, a pilot study by Lin et al. (2023) combining rTMS with transspinal electrical stimulation in individuals with incomplete SCI demonstrated enhanced corticospinal excitability, supporting the feasibility of multi-level stimulation.

Despite this theoretical and preliminary evidence, no controlled clinical trial has evaluated the combined application of rTMS (or iTBS) and tSCS for upper limb recovery in cervical SCI. The present protocol addresses this critical evidence gap.

### Objectives

#### Primary objective

To evaluate the feasibility, safety, and preliminary efficacy of combined iTBS and individualized tSCS, delivered with standardized upper limb rehabilitation, compared with tSCS and rehabilitation alone, for improving upper limb motor function in adults with chronic incomplete cervical SCI.

#### Secondary objectives

1. To assess the effects of adjunctive iTBS on UE functional capacity, independence in activities of daily living, and health-related quality of life.

2. To characterize the neurophysiological effects of combined stimulation on corticospinal excitability, including central motor conduction time and motor evoked potential parameters.

3. To evaluate the safety and tolerability of the combined intervention.

4. To determine the durability of treatment effects at 4- and 12-week follow-up.

5. To generate effect size estimates and feasibility data to inform the design of a subsequent adequately powered multicenter confirmatory trial.

#### Hypotheses

We hypothesize that participants receiving adjunctive iTBS will demonstrate (1) a clinically meaningful additional improvement of 2 or more points in UEMS compared with tSCS-based rehabilitation alone, and (2) enhanced corticospinal tract plasticity, as reflected by neurophysiological measures, with functional gains maintained at follow-up. A 2-point UEMS change approximates the minimal clinically important difference in chronic cervical SCI, reflecting gain of at least one muscle grade in a key upper limb muscle and typically corresponding to a functional gain such as independent self-feeding or grooming.

## Methods

### Trial design

This is a single-center, two-arm, parallel-group, assessor-blinded, pilot randomized controlled trial. The protocol has been developed in accordance with the Standard Protocol Items: Recommendations for Interventional Trials (SPIRIT) 2013 statement (Chan et al., 2013), the SPIRIT-iNeurostim extension for neurostimulation trials (Bresnahan et al., 2024), and the CONSORT 2010 extension for pilot and feasibility trials (Eldridge et al., 2016). The trial will be prospectively registered on ClinicalTrials.gov prior to enrollment of the first participant. This protocol is reported according to JMIR Research Protocols guidelines for protocol papers.

### Ethical considerations

This protocol will be submitted for approval to the National Healthcare Group Domain Specific Review Board (NHG DSRB), which serves as the ethics committee for studies conducted at Alexandra Hospital under the National University Health System (NUHS). All participants will provide written informed consent following a comprehensive explanation of the study procedures, risks, potential benefits, and alternatives. The informed consent process will include verification of participant understanding using a teach-back method. Participants will be free to withdraw at any time without affecting their standard clinical care. The study will be conducted in accordance with the Declaration of Helsinki (2013 revision), the International Council for Harmonization Good Clinical Practice (ICH-GCP) guidelines, and Singapore’s Human Biomedical Research Act (HBRA).

### Study setting

The trial will be conducted at the. Alexandra Hospital is a public general and rehabilitation hospital that provides comprehensive inpatient and outpatient SCI rehabilitation services under the NUHS cluster. All study interventions and assessments will take place in a dedicated neuromodulation research suite within the rehabilitation department.

### Eligibility criteria

The inclusion and exclusion criteria are summarized in Table 1.

**TABLE 1:** Eligibility criteria.

Inclusion criteria	
**Age**	21–65 years
**Chronicity**	More than 12 months post-injury at enrollment
**Injury type**	Traumatic or non-traumatic incomplete cervical SCI, neurological level C2 to C8
**AIS classification**	Grade C or D
**UEMS**	10 to 20 out of 25 on the more impaired side; lower bound 10 ensures sufficient voluntary activation for task training and elicitable MEPs, upper bound avoids ceiling effects.
**Grip strength**	MMT grade 3 or higher in finger flexors (C8 myotome) on at least one side.
**Hand function**	Able to transfer at least 1 block across the partition within 60 s on the more impaired side.
**Sitting tolerance**	Able to tolerate upright seated posture in own wheelchair for at least 1 continuous hour without symptomatic orthostatic hypotension or pressure-related discomfort requiring position change.
**Medical stability**	No acute medical complications
**Informed consent**	Able to provide written informed consent and comply with the study schedule
**Medications**	Stable regimen for 4 weeks prior, AND participant plus clinician agreement that dosage (baclofen, tizanidine, botulinum toxin) remains unchanged throughout the 12-week intervention; any change logged as protocol deviation
**Surgical clearance**	Cleared by neurosurgeon or orthopedic surgeon for participation in tSCS-based rehabilitation
Exclusion criteria	
**Seizure history**	History of seizures or epilepsy
**Implanted devices**	Intracranial metallic implants, cochlear implants, cardiac pacemakers, or other implanted electronic devices. Cervical spinal instrumentation (e.g., posterior rods, plates, or screws at C2 to T1) is not an exclusion for tSCS provided overlying skin is intact; participants with hardware directly beneath planned electrode sites will undergo low-intensity test stimulation during screening, and electrode placement will be shifted by one interspace if current distortion, focal discomfort, or unexpected motor thresholds are observed. Intracranial hardware remains an absolute exclusion for iTBS.
**Prior craniotomy**	Prior neurosurgical procedure involving craniotomy
**Pregnancy**	Currently pregnant or intending to become pregnant during the study period
**Psychiatric or cognitive**	Active psychiatric illness (eg, untreated major depression, psychosis) or cognitive impairment precluding informed consent
**Concurrent neurological disease**	Progressive or degenerative neurological condition (eg, multiple sclerosis, motor neuron disease)
**Concurrent trials**	Participation in another interventional rehabilitation or neurostimulation trial
**Skin integrity**	Skin lesions or breakdown at electrode placement sites (scalp or posterior cervical spine)
**Cortical excitability medications**	Medications known to alter cortical excitability (eg, antiepileptic drugs, high-dose benzodiazepines) that cannot be stabilized for at least 2 weeks prior to enrollment
**Severe spasticity**	Modified Tardieu Scale muscle reaction grade 4 (unfatigable clonus > 10 s) at elbow or wrist flexors on the more impaired side, or spasticity judged by the treating physician as unresponsive to optimized pharmacological management.

AIS, American Spinal Injury Association Impairment Scale; UEMS, Upper Extremity Motor Score; SCI, spinal cord injury; tSCS, transcutaneous spinal cord stimulation.

Restriction to motor-incomplete injuries is mechanistic. Convergent neuromodulation relies on residual corticospinal substrate for STDP-driven strengthening; AIS A and motor-absent AIS B injuries cannot reliably engage this pathway and would produce UEMS floor effects. Extension to AIS B participants with preserved transcranial MEPs is planned for the confirmatory trial.

### Recruitment and retention

Participants will be recruited from the outpatient SCI clinic and inpatient rehabilitation ward at Alexandra Hospital and National University Hospital, as well as through the SCI patient registry maintained by the NUHS rehabilitation service, community SCI support organizations (e.g., SPD, Singapore), and social media advertisements. Strategies to optimize recruitment and reduce dropout include flexible scheduling of treatment sessions, provision of make-up sessions for missed appointments (up to 2 per month), reimbursement of travel expenses, and regular communication with participants regarding study progress. A recruitment rate of 2–3 participants per month is anticipated based on institutional patient volumes.

### Randomization and allocation concealment

Participants will be randomized in a 1:1 ratio using a computer-generated, permuted block randomization schedule (block sizes of 4 and 6, randomly varied) stratified by AIS grade (C versus D) and baseline UEMS on the more impaired side (10–14 versus 15–20). The randomization schedule will be generated by an independent biostatistician at the Clinical Research & Innovation Office, Tan Tock Seng Hospital, with no direct involvement in participant recruitment or assessment, and stored in a secure, web-based centralized randomization system (REDCap Randomization module). Allocation concealment will be ensured by sequential, opaque, sealed envelopes accessible only to the unblinded intervention therapist.

### Blinding

This trial employs an assessor-blinded design in which outcome assessors are blinded to treatment allocation. A dedicated, unblinded intervention therapist will be responsible for delivering iTBS and configuring tSCS at each session and will have no involvement in outcome assessment. Participants in the control arm will be aware that they are not receiving iTBS, as the brief nature of the iTBS procedure and the characteristic scalp sensation make participant-level sham impractical in this pilot study. To mitigate expectation effects, both arms will be framed during consent as “active neuromodulation combined with rehabilitation” with no comparative ranking. Both groups receive identical therapist contact time, session structure, and rehabilitation content. The Credibility/Expectancy Questionnaire will be administered at baseline and week 2 and entered as a covariate in sensitivity analyses of the primary outcome. However, assessor blinding will be rigorously maintained through physical separation of intervention delivery and outcome assessment areas within the rehabilitation department. The success of assessor blinding will be formally evaluated using a blinding questionnaire at week 12 and week 24. Bang’s Blinding Index will be used, a validated metric ranging from minus 1 (opposite guessing) to +1 (complete unblinding), with 0 indicating random guessing. If Bang’s Blinding Index exceeds 0.2 with 95% CI excluding zero, a pre-planned sensitivity analysis will re-analyze the primary outcome using only objective/instrumented measures (grip/pinch dynamometry, Nine-Hole Peg Test, Box and Block Test, and neurophysiological parameters), and an independent second assessor will re-score a random 30% sample of ISNCSCI examinations from video recordings.

### Interventions

#### Overview

Both arms will receive individualized tSCS combined with standardized upper limb rehabilitation. The experimental arm will additionally receive iTBS immediately prior to each rehabilitation session. Interventions will be delivered twice per week for 12 weeks, yielding a total of 24 sessions. A minimum of 20 completed sessions (83%) will be required for per-protocol analysis.

#### Transcutaneous spinal cord stimulation protocol (both arms)

Individualized tSCS will be delivered using a programmable constant-current stimulator (DS8R, Digitimer, UK, or equivalent) with 5 × 10 cm self-adhesive PALS electrodes (Axelgaard, USA) via adhesive electrodes placed over the posterior midline of the cervical spine at the spinous process interspaces one level above and below the participant’s injury level (typically C3-C4 and/or C6-C7), targeting the dorsal roots corresponding to the upper limb motor pools (C5 to T1). This placement aligns with segment-specific dorsal root activation demonstrated by Sayenko et al. (2015) and the cervical programming recommendations of Gelenitis et al. (2025). Reference electrodes will be placed bilaterally over the anterior superior iliac spines (ASIS) or clavicles, selected based on individual tolerance and response (Gelenitis et al., 2025). Stimulation parameters will be as follows: biphasic rectangular pulses at 30 Hz burst frequency, each 1 ms pulse filled with a 10 kHz carrier frequency, intensity individualized between 40 and 120 mA. The 10 kHz carrier reduces cutaneous nociceptor activation and permits the higher intensities required for deep dorsal root activation at cervical levels. These parameters are consistent with those employed in the Up-LIFT trial and the published programming recommendations (Gelenitis et al., 2025; Moritz et al., 2024). Intensities up to 120 mA at the cervical level have been demonstrated as safe and tolerable in Up-LIFT (Moritz et al., 2024) and Gelenitis et al. (2025) when delivered with a 10 kHz carrier; cardiovascular safety at this intensity range was confirmed in Samejima et al. (2025). Stimulation will be delivered continuously during the rehabilitation session (approximately 45 min). Titration algorithm: (1) verify electrode placement and skin impedance; (2) ramp intensity in 5 mA increments from 20 mA; (3) identify motor response threshold (visible or palpable muscle activation); (4) set therapeutic intensity at 80–90% of tolerance threshold or intensity eliciting motor facilitation without discomfort; (5) re-titrate every 4 sessions to account for habituation.

#### Intermittent theta burst stimulation protocol (experimental arm only)

iTBS will be delivered before each tSCS and rehabilitation session using a figure-of-eight coil connected to a biphasic magnetic stimulator (MagPro X100 with MC-B70 figure-of-eight coil, MagVenture, Denmark, or equivalent). The coil will be positioned over the hand representation area of the primary motor cortex (M1) contralateral to the more impaired upper limb. The motor hotspot will be determined as the scalp location yielding the largest and most consistent motor evoked potentials (MEPs) in the first dorsal interosseous (FDI) or abductor pollicis brevis (APB) muscle. The active motor threshold (AMT) will be determined using the relative frequency method as the minimum stimulator intensity eliciting discernible MEPs in at least 5 of 10 consecutive trials during slight voluntary contraction (approximately 10% of maximum).

The iTBS protocol will consist of bursts of 3 magnetic pulses delivered at 50 Hz, repeated at a theta frequency of 5 Hz, with 2-s trains repeated every 10 s for 20 repetitions, delivering a total of 600 pulses per session (Huang et al., 2005). Stimulation intensity will be set at 80% of AMT. Each iTBS session will last approximately 3–4 min and will be completed before electrode placement and tSCS activation. These parameters are consistent with established international safety guidelines for rTMS (Lefaucheur et al., 2020; Rossini et al., 2015) and with the iTBS protocols employed in recent SCI studies (Feng et al., 2023; Patel et al., 2025; Shi et al., 2026).

#### Standardized upper limb rehabilitation (both arms)

All participants will receive individualized, standardized upper limb rehabilitation during each session, commencing after iTBS delivery (in the experimental arm) and continuing throughout tSCS application. The rehabilitation program is based on the functional task practice protocol employed in the Up-LIFT trial (Moritz et al., 2024) and will be prescribed and supervised by a qualified physiotherapist and occupational therapist at Alexandra Hospital. Each session will comprise approximately 45–60 min of structured UE exercises, including the following components: (1) upper limb ergometry and gross UE movements (reaching, overhead press); (2) isolated finger movements and fine motor tasks; (3) simple and complex pinch tasks using graded objects; (4) grip strength training; (5) bimanual coordination tasks; and (6) individualized functional activities prescribed by the attending occupational therapist based on their clinical assessment. Task difficulty will be adjusted according to the participant’s functional capacity using a standardized progression algorithm.

#### Treatment session sequence

Each session in the experimental arm will follow the sequence: (1) iTBS over M1 (approximately 3–4 min); (2) tSCS electrode placement and activation (approximately 5 min setup); (3) combined tSCS plus upper limb rehabilitation (approximately 45–60 min). Total session duration for the experimental arm is approximately 55–70 min. The control arm follows the same sequence without the iTBS component, with total session duration of approximately 50–65 min.

### Outcome measures

#### Primary outcome

The primary outcome is the change in Upper Extremity Motor Score (UEMS) from baseline (week 0) to post-intervention (week 12). The UEMS is a component of the International Standards for Neurological Classification of Spinal Cord Injury (ISNCSCI) examination and assesses the strength of 10 key muscles of the upper extremities (5 per side) on a 0 to 5 scale, yielding a score of 0 to 25 per side or 0 to 50 bilaterally. The UEMS has well-established psychometric properties, is sensitive to change in SCI populations, and was used as a primary or secondary endpoint in the Up-LIFT trial and other major SCI neuromodulation studies (Moritz et al., 2024; Singh et al., 2024).

#### Secondary outcomes

Secondary clinical and neurophysiological outcomes are summarized in Table 2.

**TABLE 2:** Secondary outcome measures.

Outcome measure	Description	Domain	Timepoints
GRASSP-2	Graded Redefined Assessment of Strength, Sensibility and Prehension version 2; comprehensive UE impairment measure for tetraplegia	Motor/Sensory	Wk 0, 12, 16, 24
Grip/pinch strength	Maximal grip force (Jamar dynamometer), lateral pinch, tip pinch, and tripod pinch (pinch gauge)	Motor	Wk 0, 12, 16, 24
Nine-Hole Peg Test	Timed fine motor dexterity assessment	Fine Motor	Wk 0, 12, 16, 24
Box and Block Test	Gross manual dexterity (blocks transferred in 60 s)	Gross Motor	Wk 0, 12, 16, 24
CUE-T	Capabilities of Upper Extremity Test; objective functional assessment for tetraplegia	Function	Wk 0, 12, 16, 24
SCIM-III	Spinal Cord Independence Measure III; self-care, respiration, sphincter management, mobility	Independence	Wk 0, 12, 16, 24
Modified Tardieu Scale	Velocity-dependent spasticity of elbow and wrist flexors/extensors	Spasticity	Wk 0, 6, 12, 16, 24
CMCT	CMCT per IFCN guidelines: CMCT = MEP latency minus PCT, where PCT = (F-latency + M-latency minus 1) / 2	Neurophysiology	Wk 0, 12, 16, 24
MEP parameters	Amplitude, latency, resting and active motor thresholds from FDI and APB	Neurophysiology	Wk 0, 12, 16, 24
EMG	Surface electromyography from key upper limb muscles during standardized tasks	Neurophysiology	Wk 0, 12, 16, 24
EQs-5D-5L	Generic health-related quality of life; utility index and visual analog scale	Quality of Life	Wk 0, 12, 24
Modified GAS	Patient-identified goals scored on a standardized attainment scale	Patient-Centered	Wk 0, 12, 16, 24
Adverse events	Structured adverse event recording at every session	Safety	Every session

GRASSP-2, Graded Redefined Assessment of Strength, Sensibility and Prehension version 2; CUE-T, Capabilities of Upper Extremity Test; SCIM-III, Spinal Cord Independence Measure III; CMCT, central motor conduction time; MEP, motor evoked potential; FDI, first dorsal interosseous; APB, abductor pollicis brevis; EMG, electromyography; GAS, Goal Attainment Scale; Wk, week.

##### Feasibility outcomes

As this is a pilot study, pre-specified feasibility outcomes will be evaluated against the following progression criteria: (1) recruitment rate of at least 2 participants per month; (2) retention rate of 80% or higher (i.e., at least 20 of 24 participants completing the study); (3) intervention adherence of 83% or higher (i.e., at least 20 of 24 sessions completed); (4) assessment completion rate of 90% or higher for the primary outcome; and (5) no serious adverse events attributable to the combined intervention. These criteria will determine whether progression to a full-scale confirmatory trial is warranted.

### Study schedule

#### Data collection and management

The schedule of enrollment, interventions, and assessments is presented in Table 3. Clinical outcomes will be assessed by trained, certified assessors who are blinded to group allocation and have demonstrated inter-rater reliability (intraclass correlation coefficient of 0.85 or higher) on the ISNCSCI examination. All data will be collected using electronic case report forms (eCRFs) hosted on a secure REDCap database maintained by the Clinical Research & Innovation Office, Tan Tock Seng Hospital, with automated range checks, audit trails, and role-based access control. Neurophysiological data (MEPs, CMCT, EMG) will be recorded using a dedicated EMG system and processed semi-automatically with manual verification by a blinded neurophysiologist. All data will be backed up daily and stored on encrypted institutional servers in compliance with Singapore’s Personal Data Protection Act (PDPA) and the HBRA.

**TABLE 3:** SPIRIT schedule of enrollment, interventions, and assessments.

Timepoint	Screen	Baseline (Wk 0)	Intervention (Wk 1-12)	Post-Tx (Wk 12)	FU1 (Wk 16)	FU2 (Wk 24)
Informed consent	X					
Eligibility assessment	X					
Medical history	X					
Surgeon clearance	X					
Randomization		X				
iTBS (experimental arm)			2x/wk			
tSCS (both arms)			2x/wk			
UL rehabilitation (both arms)			2x/wk Wk 6 Each visit			
ISNCSCI / UEMS		X		X	X	X
GRASSP-2		X		X	X	X
Grip/Pinch strength		X		X	X	X
Nine-Hole Peg Test		X		X	X	X
Box and Block Test		X		X	X	X
CUE-T		X		X	X	X
SCIM-III		X		X	X	X
Modified Tardieu Scale		X		X	X	X
CMCT and MEP assessment		X		X	X	X
EMG		X		X	X	X
EQ-5D-5L		X		X		X
Modified GAS		X		X	X	X
Adverse events				X	X	X
Blinding assessment				X		X

ISNCSCI, International Standards for Neurological Classification of Spinal Cord Injury; UEMS, Upper Extremity Motor Score; GRASSP-2, Graded Redefined Assessment of Strength, Sensibility and Prehension version 2; CUE-T, Capabilities of Upper Extremity Test; SCIM-III, Spinal Cord Independence Measure III; CMCT, central motor conduction time; MEP, motor evoked potential; EMG, electromyography; GAS, Goal Attainment Scale; FU, follow-up; Wk, week; UL, upper limb.

#### Sample size

This is an exploratory pilot trial, and the sample size of 24 participants (12 per group) is based primarily on feasibility considerations and is consistent with recommendations for pilot studies in neuromodulation and SCI rehabilitation (Julious, 2005). This sample size is sufficient to estimate the standard deviation of the primary outcome, assess feasibility parameters, and generate preliminary effect size estimates with reasonable precision to inform the power calculation for a subsequent confirmatory trial. Assuming 20% attrition, we aim to enroll 24 participants to ensure at least 10 evaluable participants per arm. This is consistent with sample sizes in comparable neuromodulation pilot trials in SCI (Feng et al., 2023; Gharooni et al., 2018; Lin et al., 2023).

### Statistical analysis

All analyses will follow the intention-to-treat (ITT) principle, defined as all randomized participants who receive at least one treatment session and have at least one post-baseline assessment. Continuous variables will be summarized as mean and standard deviation (or median and interquartile range where appropriate). Between-group comparisons for the primary outcome (UEMS change from baseline to week 12) will be analyzed using a linear mixed-effects model with treatment group, time, and treatment-by-time interaction as fixed effects, stratification factors (AIS grade and baseline UEMS) as covariates, and participant as a random effect. An unstructured covariance matrix will be assumed for repeated measures.

Secondary continuous outcomes will be analyzed using analogous mixed-effects models. Effect sizes (Cohen d) and 95% confidence intervals will be reported for all between-group comparisons to support the planning of a future definitive trial. A per-protocol analysis (participants completing 20 or more of 24 sessions) will be conducted as a sensitivity analysis. Missing data will be handled within the mixed-effects framework under the assumption of missing at random (MAR). Feasibility outcomes will be reported descriptively against the pre-specified progression criteria. A sensitivity analysis excluding participants with antispasticity dose changes will be conducted for the Modified Tardieu Scale outcome. Safety outcomes will be tabulated by treatment group with frequencies and proportions. All analyses will be performed using R (version 4.3 or later), with statistical significance set at P < .05. All between-group efficacy comparisons (primary and secondary) will be reported as exploratory. The trial’s primary purpose is estimation of effect sizes, variability, and feasibility parameters for a definitive trial. Secondary functional and neurophysiological outcomes carry elevated Type I error risk due to multiple testing without correction and should be interpreted as hypothesis-generating only.

### Data monitoring and safety

An independent Data and Safety Monitoring Committee (DSMC), comprising a biostatistician, a rehabilitation physician, and a neurologist external to the study team, will review safety data after enrollment of the first 8 and 16 participants. Pre-specified stopping rules based on serious adverse event rates will be employed. Both tSCS and iTBS are generally well tolerated, with reported adverse events limited to transient skin irritation, paresthesia, mild muscle twitching, scalp discomfort, or headache (Lefaucheur et al., 2020; Moritz et al., 2024; Rossini et al., 2015). The risk of seizure with iTBS is very low (less than 0.1%) when appropriate screening and stimulation parameters are used (Lefaucheur et al., 2020; Rossini et al., 2015). Emerging studies combining spinal and cortical neuromodulation have not reported an increased incidence of adverse events, supporting the feasibility of combined application under careful monitoring (Lin et al., 2023; Pulverenti et al., 2022). The cardiovascular safety of cervical tSCS has been established in a secondary analysis of the Up-LIFT trial involving 60 participants, which showed that tSCS combined with rehabilitation did not adversely alter blood pressure regulation (Samejima et al., 2025).

## Results

This protocol will be submitted for ethical approval to the National Healthcare Group Domain Specific Review Board (NHG DSRB). Recruitment is expected to begin in the third quarter of 2026 at Alexandra Hospital, Singapore. Data collection is anticipated to be completed by the fourth quarter of 2027, with results expected to be published by mid-2028. As of the date of manuscript preparation, no participants have been enrolled.

## Discussion

### Principal considerations

This protocol describes the first controlled clinical trial designed to evaluate the combined application of cortical neuromodulation (iTBS) and spinal neuromodulation (tSCS), a convergent multi-level stimulation strategy, for upper limb recovery in chronic incomplete cervical SCI. The rationale is grounded in the complementary mechanisms of these two non-invasive modalities: iTBS enhances descending cortical drive by increasing excitability of the primary motor cortex and corticospinal projections above the injury, while tSCS primes sublesional spinal circuits by activating dorsal root afferents and raising the baseline excitability of motoneuron pools and spinal interneuronal networks below the injury (Fan et al., 2025; Singh et al., 2024). When these two sources of facilitation converge on alpha motoneurons and spinal interneurons, particularly during active task-specific training, the conditions for spike-timing-dependent plasticity and associative strengthening of residual corticospinal connections may be optimized.

### Comparison with prior work

The use of iTBS rather than conventional high-frequency rTMS represents a deliberate design choice informed by recent evidence and practical considerations. The standard iTBS protocol delivers 600 pulses in approximately 3–4 min, compared with 10–20 min for conventional 20 Hz rTMS, significantly reducing session burden and facilitating integration into clinical rehabilitation workflows. Recent trials by Feng et al. (2023) and Shi et al. (2026) have demonstrated the efficacy of iTBS in SCI populations, and the Patel et al. (2025) protocol, also published in JMIR Research Protocols, provides further methodological precedent for iTBS-based studies in this population.

The choice of a two-arm design (combined versus tSCS alone) rather than a four-arm factorial design reflects the pilot nature of this study. While a factorial design would enable disentangling of individual and combined effects, the sample size required would be prohibitive for a first-in-class pilot. The present design is optimized to address the pragmatic clinical question of whether adding iTBS to tSCS-based rehabilitation provides incremental benefit, which is the most relevant question for clinical decision-making in settings where tSCS is increasingly being implemented. Data from this pilot will inform whether a larger factorial trial is warranted.

We acknowledge that activity-based restorative therapy literature typically favors 3–5 sessions per week, and that the 3–4 day inter-session gaps in our protocol may attenuate the cumulative spike-timing-dependent priming effects that denser schedules could achieve. This introduces a risk of Type II error should the intervention prove effective only at higher densities. We have retained the twice-weekly schedule because (i) the total dose of 24 sessions matches the Up-LIFT trial, enabling cross-study comparison; (ii) iTBS after-effects on cortical excitability persist beyond single sessions and may partially bridge inter-session gaps; (iii) feasibility and retention are primary pilot objectives; and (iv) most participants in our outpatient setting rely on caregiver-assisted transport, making thrice-weekly attendance impractical. Session density will be explicitly re-evaluated as a design parameter for the subsequent confirmatory trial, and pilot effect-size estimates should be interpreted as a lower bound on achievable benefit. The inclusion of a 12-week post-intervention follow-up extends beyond the single 4-week follow-up used in some prior studies, providing more robust data on durability of treatment effects.

### Strengths

Several methodological strengths distinguish this protocol. First, the comprehensive outcome battery spans multiple domains of the International Classification of Functioning, Disability and Health (ICF) framework: motor impairment (UEMS, GRASSP-2, grip/pinch strength), fine and gross motor dexterity (Nine-Hole Peg Test, Box and Block Test), functional capacity (CUE-T), independence (SCIM-III), spasticity (Modified Tardieu Scale), neurophysiology (CMCT, MEPs, EMG), quality of life (EQ-5D-5L), and patient-centered goals (Modified GAS). Second, the inclusion of CMCT and MEP assessments provides mechanistic insight into whether the combined intervention produces measurable changes in corticospinal tract integrity and excitability. Third, the standardized tSCS protocol, informed by the largest published dataset on cervical tSCS programming (Gelenitis et al., 2025), enhances reproducibility and comparability with the existing evidence base. Fourth, the inclusion of the EQ-5D-5L enables future health economic evaluation of the combined intervention. Fifth, the study is situated in a Southeast Asian rehabilitation center, extending the evidence base beyond North American and European settings where the majority of tSCS research has been conducted.

### Limitations

Several limitations should be acknowledged. First, the pilot sample size of 24 participants (12 per group) limits statistical power for detecting small to moderate between-group differences and precludes definitive efficacy conclusions. This is mitigated by the explicit pilot framing and the pre-specified feasibility progression criteria. Second, the single-center design at Alexandra Hospital may limit generalizability, although it enhances protocol fidelity and reduces inter-site variability. Third, the assessor-blinded (rather than double-blinded) design introduces the possibility of performance bias, as participants in the experimental arm are aware of receiving iTBS. However, assessor blinding will be rigorously maintained, and all primary and secondary outcomes are either clinician-assessed or objective measures. Fourth, the heterogeneity inherent in the SCI population, including variations in injury level, completeness, and chronicity, may introduce variability in treatment responses; the stratified randomization is designed to address this concern. Fifth, the absence of a standalone iTBS arm means that the independent contribution of iTBS cannot be isolated from its interaction with tSCS, although this was a deliberate design choice to maximize the clinical relevance of the comparison for a pilot study.

## Conclusion

This pilot randomized controlled trial will provide the first controlled evidence on whether adjunctive cortical neuromodulation via iTBS enhances upper limb motor recovery beyond tSCS-based rehabilitation alone in individuals with chronic incomplete cervical SCI. The feasibility data, effect size estimates, and safety profile generated will directly inform the design and powering of a subsequent multicenter confirmatory trial. By targeting both supraspinal and spinal levels of the motor neuraxis with non-invasive stimulation alongside structured rehabilitation, this convergent neuromodulation approach has the potential to advance the therapeutic landscape for individuals with tetraplegia who face substantial unmet clinical need.

## References

[B1] Ahuja C. S. Wilson J. R. Nori S. Kotter M. R. N. Druschel C. Curt A. et al. (2017). Traumatic spinal cord injury. *Nat. Rev. Dis. Primers* 3:17018. 10.1038/nrdp.2017.18 28447605

[B2] Anderson K. D. (2004). Targeting recovery: Priorities of the spinal cord-injured population. *J. Neurotrauma* 21 1371–1383. 10.1089/neu.2004.21.1371 15672628

[B3] Anderson M. A. Squair J. W. Gautier M. Hutson T. H. Kathe C. Barraud Q. et al. (2022). Natural and targeted circuit reorganization after spinal cord injury. *Nat. Neurosci.* 25 1584–1596. 10.1038/s41593-022-01196-1 36396975

[B4] Barss T. S. Parhizi B. Porter J. Mushahwar V. K. (2022). Neural substrates of transcutaneous spinal cord stimulation: Neuromodulation across multiple segments of the spinal cord. *J. Clin. Med.* 11:639. 10.3390/jcm11030639 35160091 PMC8836636

[B5] Behrman A. L. Bowden M. G. Nair P. M. (2006). Neuroplasticity after spinal cord injury and training: An emerging paradigm shift in rehabilitation and walking recovery. *Phys. Ther.* 86 1406–1425. 10.2522/ptj.20050212 17012645

[B6] Benavides F. D. Jo H. J. Lundell H. Edgerton V. R. Gerasimenko Y. Perez M. A. (2020). Cortical and subcortical effects of transcutaneous spinal cord stimulation in humans with tetraplegia. *J. Neurosci.* 40 2633–2643. 10.1523/jneurosci.2374-19.2020 31996455 PMC7096150

[B7] Bresnahan R. Copley S. Eldabe S. Thomson S. North R. B. Baranidharan G. et al. (2024). Reporting guidelines for protocols of randomised controlled trials of implantable neurostimulation devices: The SPIRIT-iNeurostim extension. *EClinicalMedicine* 78:102933. 10.1016/j.eclinm.2024.102933 39606687 PMC11600657

[B8] Chan A. W. Tetzlaff J. M. Altman D. G. Laupacis A. Gøtzsche P. C. Krleža-Jerić K. et al. (2013). SPIRIT 2013 statement: Defining standard protocol items for clinical trials. *Ann. Intern. Med.* 158 200–207. 10.7326/0003-4819-158-3-201302050-00583 23295957 PMC5114123

[B9] Dietz V. Fouad K. (2014). Restoration of sensorimotor functions after spinal cord injury. *Brain* 137(Pt 3), 654–667. 10.1093/brain/awt262 24103913

[B10] Eldridge S. M. Chan C. L. Campbell M. J. Bond C. M. Hopewell S. Thabane L. et al. (2016). CONSORT 2010 statement: Extension to randomised pilot and feasibility trials. *BMJ* 355:i5239. 10.1136/bmj.i5239 27777223 PMC5076380

[B11] Fan S. Wang W. Zheng X. (2025). Repetitive transcranial magnetic stimulation for the treatment of spinal cord injury: Current status and perspective. *Intern. J. Mol. Sci.* 26:825. 10.3390/ijms26020825 39859537 PMC11766194

[B12] Feng X. Wang T. Jiang Y. Liu Y. Yang H. Duan Z. et al. (2023). Cerebral theta-burst stimulation combined with physiotherapy in patients with incomplete spinal cord injury: A pilot randomized controlled trial. *J. Rehabil. Med.* 55:jrm00375. 10.2340/jrm.v55.4375 36779636 PMC9941982

[B13] Gelenitis K. Santamaria A. Pradarelli J. Rieger M. Inanici F. Tefertiller C. et al. (2025). Non-invasive transcutaneous spinal cord stimulation programming recommendations for the treatment of upper extremity impairment in tetraplegia. *Neuromodulation* 28 162–173. 10.1016/j.neurom.2024.05.005 38958629

[B14] Gerasimenko Y. Gorodnichev R. Puhov A. Moshonkina T. Savochin A. Selionov V. et al. (2015). Initiation and modulation of locomotor circuitry output with multisite transcutaneous electrical stimulation of the spinal cord in noninjured humans. *J. Neurophysiol.* 113 834–842. 10.1152/jn.00609.2014 25376784

[B15] Gharooni A. A. Nair K. P. S. Hawkins D. Scivill I. Hind D. Hariharan R. (2018). Intermittent theta-burst stimulation for upper-limb dysfunction and spasticity in spinal cord injury: A single-blind randomized feasibility study. *Spinal Cord* 56 762–768. 10.1038/s41393-018-0152-5 29895874

[B16] Hofstoetter U. S. Freundl B. Danner S. M. Krenn M. J. Mayr W. Binder H. et al. (2020). Transcutaneous spinal cord stimulation induces temporary attenuation of spasticity in individuals with spinal cord injury. *J. Neurotrauma* 37 481–493. 10.1089/neu.2019.6588 31333064

[B17] Hofstoetter U. S. Freundl B. Lackner P. Binder H. (2021). Transcutaneous spinal cord stimulation enhances walking performance and reduces spasticity in individuals with multiple sclerosis. *Brain Sci.* 11:472. 10.3390/brainsci11040472 33917893 PMC8068213

[B18] Huang Y. Z. Edwards M. J. Rounis E. Bhatia K. P. Rothwell J. C. (2005). Theta burst stimulation of the human motor cortex. *Neuron* 45 201–206. 10.1016/j.neuron.2004.12.033 15664172

[B19] Hutson T. H. Di Giovanni S. (2019). The translational landscape in spinal cord injury: Focus on neuroplasticity and regeneration. *Nat. Rev. Neurol.* 15 732–745. 10.1038/s41582-019-0280-3 31728042

[B20] James N. D. McMahon S. B. Field-Fote E. C. Bradbury E. J. (2018). Neuromodulation in the restoration of function after spinal cord injury. *Lancet Neurol.* 17 905–917. 10.1016/s1474-4422(18)30287-4 30264729

[B21] Julious S. A. (2005). Sample size of 12 per group rule of thumb for a pilot study. *Pharmaceut. Statist.* 4 287–291. 10.1002/pst.185

[B22] Lefaucheur J. P. Aleman A. Baeken C. Benninger D. H. Brunelin J. Di Lazzaro V. et al. (2020). Evidence-based guidelines on the therapeutic use of repetitive transcranial magnetic stimulation (rTMS): An update (2014-2018). *Clin. Neurophysiol.* 131 474–528. 10.1016/j.clinph.2019.11.002 31901449

[B23] Lin B. S. Zhang Z. Peng C. W. Chen S. H. Chan W. P. Lai C. H. (2023). Effectiveness of repetitive transcranial magnetic stimulation combined with transspinal electrical stimulation on corticospinal excitability for individuals with incomplete spinal cord injury: A pilot study. *IEEE Trans. Neural Syst. Rehabil. Eng.* 31 4790–4800. 10.1109/tnsre.2023.3338226 38032783

[B24] Lu Y. Shang Z. Zhang W. Hu X. Shen R. Zhang K. et al. (2025). Global, regional, and national burden of spinal cord injury from 1990 to 2021 and projections for 2050: A systematic analysis for the Global Burden of Disease 2021 study. *Ageing Res. Rev.* 103:102598. 10.1016/j.arr.2024.102598 39603465

[B25] Martino Cinnera A. Bonanno M. Calabrò R. S. Bisirri A. D’Arienzo M. D’Acunto A. et al. (2024). Paired associative stimulation to enhance motor outcome in spinal cord injury: A systematic review of first evidence. *Exp. Rev. Med. Devices* 21 507–518. 10.1080/17434440.2024.2358048 38768088

[B26] Mertz L. (2025). FDA-Cleared noninvasive spine stimulation system could transform spinal cord injury treatment. *IEEE Pulse* 16 10–15. 10.1109/MPULS.2025.3572565 40668690

[B27] Moritz C. Field-Fote E. C. Tefertiller C. van Nes I. Trumbower R. Kalsi-Ryan S. et al. (2024). Non-invasive spinal cord electrical stimulation for arm and hand function in chronic tetraplegia: A safety and efficacy trial. *Nat. Med.* 30 1276–1283. 10.1038/s41591-024-02940-9 38769431 PMC11108781

[B28] Patel D. Banerjee R. Farooque K. Gupta D. Garg B. Kumar N. et al. (2025). Corticospinal intermittent theta burst stimulation propelling sensorimotor function recovery in complete spinal cord injury: Protocol for a randomized controlled trial. *JMIR Res. Protoc.* 14:e66531. 10.2196/66531 40577046 PMC12254704

[B29] Pulverenti T. S. Zaaya M. Grabowski E. Grabowski M. Knikou M. (2022). Brain and spinal cord paired stimulation coupled with locomotor training facilitates motor output in human spinal cord injury. *Front. Neurol.* 13:1000940. 10.3389/fneur.2022.1000940 36313489 PMC9612520

[B30] Pulverenti T. S. Zaaya M. Grabowski M. Grabowski E. Islam M. A. Li J. et al. (2021). Neurophysiological changes after paired brain and spinal cord stimulation coupled with locomotor training in human spinal cord injury. *Front. Neurol.* 12:627975. 10.3389/fneur.2021.627975 34040572 PMC8141587

[B31] Raineteau O. Schwab M. E. (2001). Plasticity of motor systems after incomplete spinal cord injury. *Nat. Rev. Neurosci.* 2 263–273. 10.1038/35067570 11283749

[B32] Rossini P. M. Burke D. Chen R. Cohen L. G. Daskalakis Z. Di Iorio R. et al. (2015). Non-invasive electrical and magnetic stimulation of the brain, spinal cord, roots and peripheral nerves: Basic principles and procedures for routine clinical and research application. An updated report from an I.F.C.N. Committee. *Clin. Neurophysiol.* 126 1071–1107. 10.1016/j.clinph.2015.02.001 25797650 PMC6350257

[B33] Safdarian M. Trinka E. Rahimi-Movaghar V. Thomschewski A. Aali A. Abady G. G. et al. (2023). Global, regional, and national burden of spinal cord injury, 1990–2019: A systematic analysis for the Global Burden of Disease Study 2019. *Lancet Neurol.* 22 1026–1047. 10.1016/S1474-4422(23)00287-9 37863591 PMC10584692

[B34] Samejima S. Malik R. N. Ge J. Rempel L. Cao K. Desai S. et al. (2025). Cardiovascular safety of transcutaneous spinal cord stimulation in cervical spinal cord injury. *Neurotherapeutics* 22:e00528. 10.1016/j.neurot.2025.e00528 39893085 PMC12014404

[B35] Sayenko D. G. Atkinson D. A. Dy C. J. Gurley K. M. Smith V. L. Angeli C. et al. (2015). Spinal segment-specific transcutaneous stimulation differentially shapes activation pattern among motor pools in humans. *J. Appl. Physiol.* 118 1364–1374. 10.1152/japplphysiol.01128.2014 25814642 PMC4451290

[B36] Shi S. Yao J. Cai J. (2026). Efficacy of excitatory iTBS on lower limb recovery in iSCI patients. *Global Spine J.* 16 287–296. 10.1177/21925682251343528 40459847 PMC12133774

[B37] Singh G. Sharma P. Forrest G. Harkema S. Behrman A. Gerasimenko Y. (2024). Spinal cord transcutaneous stimulation in cervical spinal cord injury: A review examining upper extremity neuromotor control, recovery mechanisms, and future directions. *J. Neurotrauma* 41 2056–2074. 10.1089/neu.2023.0438 38874496 PMC11971538

[B38] Suggitt J. Symonds J. D’Amico J. M. (2025). Safety and effectiveness of multisite transcutaneous spinal cord stimulation combined with activity-based therapy when delivered in a community rehabilitation setting: A real-world pilot study. *Neuromodulation* 28 1144–1156. 10.1016/j.neurom.2025.01.005 39998450

[B39] Taccola G. Sayenko D. Gad P. Gerasimenko Y. Edgerton V. R. (2018). And yet it moves: Recovery of volitional control after spinal cord injury. *Prog. Neurobiol.* 160 64–81. 10.1016/j.pneurobio.2017.10.004 29102670 PMC5773077

[B40] Tajali S. Balbinot G. Pakosh M. Sayenko D. G. Zariffa J. Masani K. (2024). Modulations in neural pathways excitability post transcutaneous spinal cord stimulation among individuals with spinal cord injury: A systematic review. *Front. Neurosci.* 18:1372222. 10.3389/fnins.2024.1372222 38591069 PMC11000807

[B41] Tefertiller C. Trumbower R. D. Morse L. Pradarelli J. Gelenitis K. D’Amico J. M. et al. (2025). Home-Based noninvasive spinal cord stimulation safely enhances hand and arm function in people with spinal cord injury. *Neurol. Clin. Pract.* 15:e200537. 10.1212/cpj.0000000000200537 41019097 PMC12462424

[B42] van Silfhout L. Hosman A. J. F. Bartels R. Edwards M. J. R. Abel R. Curt A. et al. (2017). Ten meters walking speed in spinal cord-injured patients: Does speed predict who walks and who rolls? *Neurorehabil. Neural Repair* 31 842–850. 10.1177/1545968317723751 28786305

[B43] Wang Y. Dong T. Li X. Zhao H. Yang L. Xu R. et al. (2023). Research progress on the application of transcranial magnetic stimulation in spinal cord injury rehabilitation: A narrative review. *Front. Neurol.* 14:1219590. 10.3389/fneur.2023.1219590 37533475 PMC10392830

[B44] Wankner M. C. Visser-Vandewalle V. Andrade P. Heiden P. (2026). Cervical spinal cord stimulation for functional rehabilitation after spinal cord injury: A systematic review of preclinical and clinical studies. *Life* 16:179. 10.3390/life16010179 41598333 PMC12843007

[B45] Zheng Y. Mao Y. R. Yuan T. F. Xu D. S. Cheng L. M. (2020). Multimodal treatment for spinal cord injury: A sword of neuroregeneration upon neuromodulation. *Neural Regen. Res.* 15 1437–1450. 10.4103/1673-5374.274332 31997803 PMC7059565

[B46] Zholudeva L. V. Qiang L. Marchenko V. Dougherty K. J. Sakiyama-Elbert S. E. Lane M. A. (2018). The neuroplastic and therapeutic potential of spinal interneurons in the injured spinal cord. *Trends Neurosci.* 41 625–639. 10.1016/j.tins.2018.06.004 30017476 PMC6109421

